# Predicting operative time in robotic ventral hernia repair using machine learning: a retrospective pilot study

**DOI:** 10.1007/s00423-026-04057-8

**Published:** 2026-04-24

**Authors:** Francesco Brucchi, Alice Gori, Ilia Van Campenhout, Jan Colpaert, Kim Boterbergh, Peter Potvlieghe, Tommaso Violante, Matteo Rottoli, Gianlorenzo Dionigi, Filip Muysoms, Bert Van Den Bossche

**Affiliations:** 1 Department of Pathophysiology and Transplantation, University of Milan, Milan, Italy; 2 Department of Medical and Surgical Sciences, University of Bologna, Bologna, Italy; 3 Orsi Academy, Melle, Belgium; 4 Department of Surgery of Alimentary Tract, IRCCS AOU di Bologna, Bologna, Italy; 5 Division of General Surgery, Istituto di Ricovero e Cura a Carattere Scientifico (IRCCS) Istituto Auxologico Italiano, Via Mercalli 30, Milan, 20122 Italy; 6 Department of General, Digestive and HPB Surgery, AZORG Hospital, Aalst, Belgium; 7 Department of General Surgery, AZ Maria Middelares, Ghent, Belgium

**Keywords:** Robotic ventral hernia repair, Operative time prediction, Machine learning, Random forest, Surgical planning, SHAP analysis

## Abstract

**Background:**

Robotic ventral hernia repair (VHR) is increasingly common, yet predicting operative time remains challenging. No study has yet applied machine learning (ML) to this setting.

**Objective:**

To compare ML models for predicting operative time in elective robotic VHR and identify key preoperative predictors.

**Methods:**

Retrospective single-center cohort study (AZORG Hospital, Aalst, Belgium) including 208 consecutive patients undergoing elective robotic VHR (October 2020–April 2025). Three models—Random Forest, Gradient Boosting, and Ridge Regression—were trained with 5-fold cross-validation using demographics, BMI, EHS hernia size, mesh position, and surgical complexity. Performance was assessed by MAE, RMSE, and R²; feature importance by SHAP analysis.

**Results:**

Median operative time was 90 min (IQR 60–130). Random Forest achieved the best performance (MAE 38.0 min, RMSE 52.8 min, R² 0.22), outperforming Gradient Boosting (R² 0.01) and Ridge Regression (R² −0.53). SHAP identified mesh position (45.0%), hernia size (30.7%), and BMI (14.2%) as the top predictors.

**Conclusion:**

In this pilot study, Random Forest provided modest operative time predictions (R² 0.22). Limited explained variance likely reflects the absence of surgeon experience and intraoperative variables. Although mesh position and hernia size are clinically consistent predictors, current accuracy is insufficient for clinical implementation. Larger multicenter studies incorporating surgeon-level data are needed.

## Introduction

The first series of robotic inguinal hernia repair (IHR) performed by a general surgeon was published in 2015 [1], marking a major milestone in surgical practice. Since then, the use of robotic platforms for both IHR and ventral hernia repair (VHR) has expanded rapidly, supported by growing evidence of their safety, effectiveness, and potential advantages over traditional surgical approaches [2–4].

Predicting operative time is essential for optimizing surgical planning and resource allocation [5–7], especially in robotic surgery, which is increasingly being adopted for VHR. While robotic surgery offers significant advantages in precision, the variability inherent in these procedures makes predicting operative time challenging. Accurate predictions can aid in efficient scheduling and improve the overall management of surgical resources, including operating room (OR) utilization and staff planning.

Machine learning (ML) algorithms have been increasingly applied to predict operative time across various surgical specialties, including bariatric surgery [8], robot-assisted general surgery [9], and orthopedic procedures [10]. However, no study to date has focused specifically on robotic abdominal wall surgery. Most of the existing literature has addressed other types of procedures [8–10] or alternative outcomes such as surgical site infections and complications [11–13]. Few studies have addressed the specific complexities of robotic VHR, which involves unique factors such as surgical technique, mesh placement strategy, and patient anatomy.

This study aims to evaluate and compare the performance of three ML models—Random Forest, Gradient Boosting, and Ridge Regression—in predicting operative time for elective robotic VHR using routinely available preoperative features. As an exploratory pilot study, we acknowledge from the outset that important determinants of operative time—most notably surgeon experience and intraoperative complications—were not available in our registry and therefore could not be included in the models. We present this work as a proof-of-concept to assess the feasibility of ML-based operative time prediction in this domain and to identify which preoperative variables carry the greatest predictive value.

## Methods

### Study design and data collection

This is a retrospective, single-center cohort study. All consecutive patients undergoing elective robotic VHR for primary and recurrent ventral hernias at the Department of General, Digestive and HPB Surgery of AZORG Hospital (Campus Merestraat, Aalst, Belgium) between October 2020 and April 2025 were identified from the institutional Hernia Registry. The robotic platforms used were Da Vinci Xi (Intuitive Surgical, Sunnyvale, CA) and Hugo™ RAS (Medtronic, Minneapolis, MN).

#### Exclusion criteria included

(1) open or laparoscopic surgical approach, (2) emergency procedures, and (3) missing or incomplete data regarding operative time or 30-day follow-up. From an initial cohort of 641 patients who underwent ventral hernia repair during the study period, 208 met the inclusion criteria and were included in the analysis.

This research was reported in accordance with the STROCSS 2021 guideline [14]. The requirement for ethical approval and informed consent was waived given the retrospective observational design of the study.

### Variable definition

Variables were selected based on findings from prior studies on operative time prediction [8, 15–17]. Patient characteristics included age, body mass index (BMI), hernia size according to the European Hernia Society (EHS) classification [18], presence of comorbidities, and hernia content. Surgical variables included mesh position (intraperitoneal, preperitoneal, or retromuscular), defect closure (yes/no), type of repair (mesh vs. suture), and requirement for intestinal resection.

Operative time was defined as the interval from the initial skin incision to the completion of all procedure-related tasks, including wound closure. This definition does not include anesthesia induction time or pre-incision preparatory steps.

Notably, several variables recognized as important determinants of operative time were not included in the model. Surgeon identity and experience level, the specific robotic platform used for each case, the presence and training level of surgical residents or trainees, anesthesia-related variables, and intraoperative events (e.g., complications, unexpected findings, adhesiolysis extent) were either not systematically recorded in the registry or could not be retrospectively retrieved for older cases. The absence of these variables represents a significant limitation of this study, as discussed below.

Categorical variables were converted into dummy variables using one-hot encoding (OHE). Additionally, three engineered binary features were created to enhance clinical interpretability:

- Large Hernia: 1 if hernia width classified as W2 or W3 (EHS), 0 otherwise.
- Obese: 1 if BMI ≥ 30 kg/m², 0 otherwise.
- Complex Content: 1 if hernia content involved omentum, intestine, or colon, 0 otherwise.

### Feature Selection

The following nine features were selected for inclusion in the predictive models based on their availability at the preoperative stage and their potential influence on operative time: age, BMI, large hernia (binary), obese (binary), complex content (binary), mesh position, defect closure (yes/no), type of repair (mesh vs. suture), and intestinal resection (yes/no).

### Machine learning models

Three ML models were evaluated: Random Forest, Gradient Boosting, and Ridge Regression. These models were selected to represent different algorithmic approaches—ensemble tree-based methods (Random Forest, Gradient Boosting) and regularized linear regression (Ridge)—to assess whether non-linear modeling provides advantages over linear approaches for this prediction task.

The dataset was randomly split into a training set (80%, *n* = 166) and an independent test set (20%, *n* = 42). Model training and hyperparameter optimization were performed on the training set using 5-fold cross-validation with grid search. The following hyperparameter spaces were explored:

- Random Forest: n_estimators = 100, max_depth = 10.
- Gradient Boosting: n_estimators = 200, max_depth = 5, learning_rate = 0.05.
- Ridge Regression: alpha = 1.0.

The optimal configurations identified by grid search were: Random Forest (n_estimators = 100, max_depth = 10), Gradient Boosting (n_estimators = 200, max_depth = 5, learning_rate = 0.05), and Ridge Regression (alpha = 1.0). Final model performance was evaluated on the held-out test set.

### Model interpretability

Feature importance for the best-performing model was assessed using the SHapley Additive exPlanations (SHAP) framework [19], which provides a unified measure of feature contribution based on cooperative game theory. SHAP values were computed on the test set to identify the most influential predictors of operative time.

### Performance evaluation

Model performance was evaluated using three metrics: mean absolute error (MAE), representing the average magnitude of prediction errors in minutes; root mean square error (RMSE), which penalizes larger errors more heavily; and the coefficient of determination (R²), indicating the proportion of variance in operative time explained by the model. An R² of 1.0 indicates perfect prediction, 0.0 indicates performance equivalent to predicting the mean, and negative values indicate performance worse than the mean baseline.

All analyses were conducted using Python 3.10 (Python Software Foundation) with the scikit-learn (v1.3), pandas (v2.0), and SHAP (v0.42) libraries.

## Results

### Patient characteristics

From the 641 patients who underwent ventral hernia repair during the study period, 208 patients undergoing elective robotic VHR were included. The demographic and surgical characteristics are summarized in Table 1. The mean age was 57 ± 16 years, 61.5% were male, and the mean BMI was 28.5 ± 5.9 kg/m². At least one comorbidity was present in 45.7% of patients. The median operative time was 90 min (IQR 60–130), with a right-skewed distribution reflecting the heterogeneity of surgical complexity.

**Table 1:** Demographic and surgical characteristics of the study cohort (*n* = 208)

Variable	Value
Age (years), mean ± SD	57 ± 16
Sex: Male, n (%)	128 (61.5%)
BMI (kg/m²), mean ± SD	28.5 ± 5.9
Presence of ≥ 1 comorbidity, n (%)	95 (45.7%)
Defect closure, n (%)	199 (95.7%)
Operative time (min), median (IQR)	90 (60–130)
Mesh position	
Intraperitoneal, n (%)	4 (1.9%)
Preperitoneal, n (%)	93 (44.7%)
Retromuscular, n (%)	111 (53.4%)
Hernia type	
Umbilical, n (%)	100 (48.1%)
Lateral, n (%)	57 (27.4%)
Epigastric, n (%)	38 (18.3%)
Parastomal, n (%)	7 (3.4%)
Spigelian, n (%)	5 (2.4%)
Diaphragmatic, n (%)	1 (0.5%)
Large hernia (EHS W2 or W3), n (%)	84 (40.4%)

*BMI *body mass index, *EHS *European Hernia Society, *IQR *interquartile range, *SD *standard deviation

Regarding hernia characteristics, 40.4% of patients had a large hernia (EHS W2 or W3). The most common hernia type was umbilical (48.3%), followed by lateral (27.5%) and epigastric (18.5%). As for mesh positioning, the majority of patients underwent retromuscular placement (53.4%), followed by preperitoneal (44.7%) and intraperitoneal (1.9%). Defect closure was performed in 95.7% of cases.

### Model performance

The performance of the three ML models is summarized in Table 2. Among the tested models, Random Forest achieved the best performance, with an RMSE of 52.8 min, MAE of 38.0 min, and R² of 0.22. This indicates that the model explained approximately 22% of the variance in operative time, with an average prediction error of 38 min. While this level of explained variance is consistent with other pilot ML studies in surgery, it is clearly insufficient for reliable clinical implementation and should be interpreted as a proof-of-concept rather than a clinically actionable tool.

**Table 2:** Performance metrics of the three machine learning models evaluated using 5-fold cross-validation

Model	RMSE (min)	MAE (min)	*R*²
Random Forest	52.8	38.0	0.22
Gradient Boosting	59.5	43.5	0.01
Ridge Regression	61.4	53.9	−0.53

*MAE *mean absolute error, *RMSE *root mean square error,R² coefficient of determination.Negative R² indicates performance worse than predicting the sample mean

Gradient Boosting showed substantially lower performance (RMSE = 59.5 min, MAE = 43.5 min, R² = 0.01), suggesting potential overfitting during training despite cross-validation. Ridge Regression performed worst (RMSE = 61.4 min, MAE = 53.9 min, R² = −0.53), with the negative R² indicating that the linear model predicted worse than a simple mean baseline, confirming that the relationship between the available preoperative features and operative time is substantially non-linear.

The modest R² value of the best-performing model (0.22) warrants contextualization. The MAE of 38 min, relative to the median operative time of 90 min, represents a relative error of approximately 42%. While this level of accuracy provides limited utility for precise minute-by-minute scheduling, it may offer value for broad OR block planning (e.g., distinguishing short procedures from longer ones). Furthermore, the low explained variance is likely attributable, at least in part, to the absence of surgeon-specific and intraoperative variables in the model (see Limitations). Importantly, we acknowledge that an R² of 0.22 is insufficient for reliable clinical implementation, as it indicates that approximately 78% of the variability in operative time remains unexplained by the current set of preoperative features.

### Feature importance

**Fig. 1:**
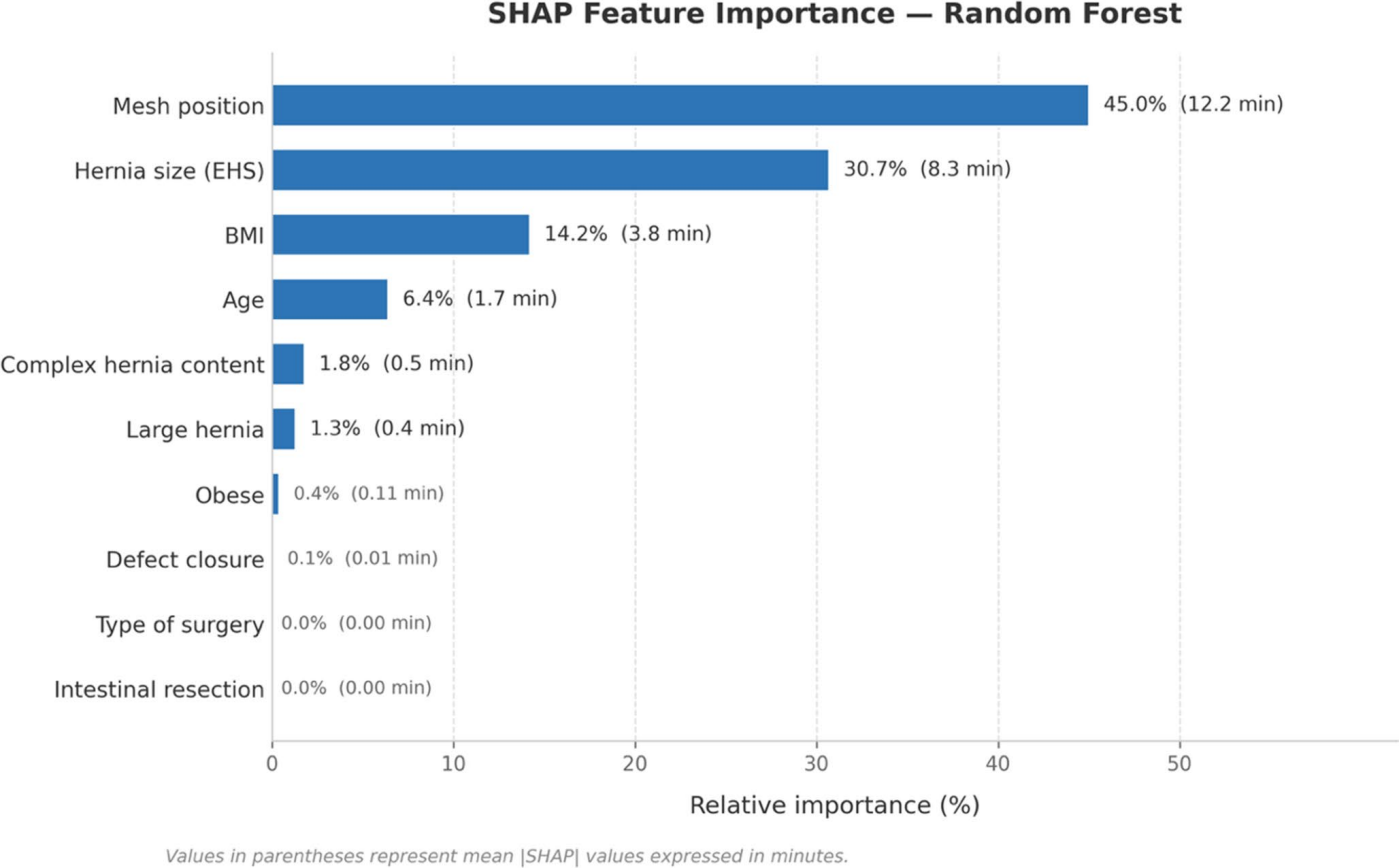
SHAP-based feature importance rankings for the Random Forest model. The horizontal bar chart displays the relative contribution (%) of each preoperative feature to the prediction of operative time. Mean absolute SHAP values (in minutes) are reported alongside relative percentages to enhance interpretability. Mesh position and hernia size (EHS classification) together account for over 75% of the model’s predictive capacity

The SHAP-based feature importance analysis for the Random Forest model is presented in Fig. 1. To improve interpretability, both relative percentages and mean absolute SHAP values (expressed in minutes) are reported. The three most influential predictors were mesh position (45.0%), hernia size based on EHS classification (30.7%), and BMI (14.2%). Together, these three features accounted for approximately 90% of the model’s predictive capacity. Age contributed 6.4%, while the remaining features—complex hernia content (1.8%), large hernia binary indicator (1.3%), obesity status (0.4%), defect closure (0.05%), type of surgery, and intestinal resection—had minimal individual contributions.

## Discussion

In settings where OR resources are limited, accurately estimating operative times is essential for optimal surgical workflow management [6, 7]. Several studies have demonstrated that ML algorithms can outperform traditional experience-based approaches in forecasting operative times, offering more reliable, data-driven solutions for surgical planning [15, 20]. To the best of our knowledge, this is the first study to apply ML approaches to robotic abdominal wall surgery for estimating operative time based on preoperative patient and procedural characteristics.

Our findings demonstrate that the Random Forest model provided the best predictions among the tested approaches, with an MAE of 38.0 min and an RMSE of 52.8 min. However, we acknowledge that the overall predictive performance is modest, as reflected by an R² of 0.22, meaning that approximately 78% of the variance in operative time was not captured by the model. When contextualized against the median operative time of 90 min (IQR 60–130), the MAE of 38 min represents a relative error of approximately 42%, which limits the model’s direct applicability for precise surgical scheduling but may still provide useful approximate estimates for broad resource planning.

These results are broadly consistent with findings from other surgical domains. Kang et al. reported RMSE values of approximately 40 min for operative time prediction in bariatric surgery using Random Forest and Gradient Boosting models [8], and other studies have reported RMSE values between 30 and 50 min across various surgical procedures [9, 10]. Our slightly higher RMSE may be attributable to the inherent variability in robotic hernia repairs, which involve factors such as heterogeneous surgeon experience, differences between robotic platforms (Da Vinci Xi vs. Hugo™ RAS), variability in surgical technique, and the wide spectrum of procedural complexity.

The superiority of the Random Forest model over Gradient Boosting and Ridge Regression in this context deserves discussion. The negative R² obtained by Ridge Regression confirms that the relationship between preoperative variables and operative time is fundamentally non-linear, and that regularized linear models are inadequate for this task. The poor performance of Gradient Boosting (R² = 0.01) despite its typically strong predictive capabilities may be related to overfitting on the relatively small training set, even with cross-validated hyperparameter tuning. Random Forest’s built-in resistance to overfitting through bootstrap aggregation (bagging) and random feature selection at each split likely explains its more robust performance in this limited-sample setting.

The SHAP analysis revealed that mesh position and hernia size were the two most influential predictors, together accounting for over 75% of the model’s feature importance. This is clinically coherent: it is well established that different mesh positions (intraperitoneal, preperitoneal, retromuscular) require substantially different surgical approaches, with the ventral transabdominal preperitoneal (TAPP) technique generally requiring less time than the extended totally extraperitoneal (e-TEP) approach and more time than intraperitoneal onlay mesh (IPOM) [21]. Larger hernias naturally demand more extensive dissection and reconstruction. BMI, the third most important feature (14.2%), likely reflects the increased technical difficulty associated with operating on patients with greater adiposity.

Interestingly, the type of repair (mesh vs. suture) and intestinal resection showed negligible feature importance. This may reflect the high degree of standardization in robotic procedures at this center, where mesh-based repair was performed in the vast majority of cases. The low contribution of the “obese” binary variable, despite BMI being an important continuous predictor, suggests that the threshold-based dichotomization (BMI ≥ 30) loses predictive information compared to the continuous BMI value, and highlights the importance of preserving continuous variables in ML models.

### Limitations

This study has several important limitations that should be carefully considered when interpreting the results, and which we believe explain the modest predictive performance observed.

First and most importantly, two of the most critical determinants of operative time—surgeon experience and intraoperative complications—were not included in the predictive models. It is well established that the surgeon’s experience level, case volume, and position on the learning curve for a specific robotic platform are among the strongest predictors of operative time in robotic surgery [22]. Similarly, intraoperative complications (e.g., bleeding, inadvertent enterotomy, dense adhesions requiring extensive adhesiolysis) can substantially prolong operative time and represent a major source of variability that is inherently unpredictable from preoperative data alone. In our registry, surgeon identity was recorded but surgeon-specific experience metrics (case volume, years of robotic experience, learning curve position) were not systematically tracked. Intraoperative complications were recorded as postoperative outcomes but not linked to operative time in a manner amenable to predictive modeling, as their occurrence is unknown before surgery. We fully acknowledge that the omission of these variables likely accounts for a significant portion of the unexplained variance (78%) in our model. Future studies must incorporate surgeon-level variables to achieve clinically meaningful predictive accuracy.

Second, this is a single-center, retrospective study with a relatively small sample size (*n* = 208). While this sample size is consistent with other pilot studies in surgical ML [8, 10], it is small for ML applications and increases the risk of overfitting, limits the generalizability of the findings to other institutions, and reduces the statistical power to detect complex variable interactions. Although 5-fold cross-validation was applied to mitigate overfitting, the risk remains non-negligible given the limited sample, and model stability cannot be guaranteed. These constraints further limit external validity and the confidence with which the results can be extrapolated to other clinical settings. The results should therefore be considered hypothesis-generating rather than definitive, and external validation on independent datasets is essential before any clinical application can be considered.

Third, the heterogeneity of robotic systems (Da Vinci Xi vs. Hugo™ RAS) used during the study period may have introduced variability that was not captured by the model, as the specific system used was not included as a feature. The two platforms differ in instrument articulation, docking procedures, and ergonomics, all of which may influence operative time. The robotic platform type therefore represents a potential confounder that may have meaningfully influenced operative duration, and its inclusion as a covariate in future models is warranted. Fourth, the model was based exclusively on preoperative variables and did not account for anesthesia-related factors (induction time, type of anesthesia) or the duration of preparatory procedures prior to incision.

Fifth, we did not compare the ML models against simpler traditional statistical approaches, such as multivariable linear or log-linear regression. The inclusion of a multivariable regression model as a benchmark is generally expected in studies evaluating ML approaches, as it provides essential context for assessing whether the added complexity of ML confers a meaningful advantage over conventional methods. Given the modest number of features (nine) and the limited sample size, it remains possible that a well-specified regression model could achieve comparable performance with greater interpretability. Including such a comparison would have strengthened the methodological rigor of the study.

Given these limitations, we wish to be transparent that we do not claim the current model is ready for clinical implementation. Rather, this study serves as an exploratory proof-of-concept demonstrating the feasibility and potential of ML-based operative time prediction in robotic abdominal wall surgery, while identifying the preoperative variables that carry the greatest predictive weight.

### Future directions

Future research should focus on several areas to address the limitations of this study. Most critically, incorporating surgeon-level variables (identity, case volume, years of experience with each robotic platform, and learning curve metrics) should be a priority, as these are expected to substantially improve model performance. Prospective study designs with standardized data collection protocols would allow systematic capture of intraoperative complications and their relationship to operative time. Multicenter studies with larger and more diverse patient populations are essential to validate the generalizability of these models. The incorporation of individual surgical step durations (dissection, suturing, mesh placement) and real-time data from robotic platforms could further enhance predictive accuracy. Importantly, although ML models represent an innovative approach that can capture complex non-linear patterns, we believe they should complement rather than replace clinical judgment [23, 24, 25]. The integration of data-driven predictions with expert human insight may offer the most robust approach to operative time estimation in clinical practice.

## Conclusion

This exploratory pilot study demonstrates that among three tested ML models, the Random Forest algorithm provided the best—albeit modest—predictions of operative time for elective robotic ventral hernia repair, with an MAE of 38 min and an R² of 0.22. Mesh position, hernia size, and BMI were identified as the most influential preoperative predictors, consistent with clinical experience. The limited explained variance likely reflects the absence of critical variables—particularly surgeon experience and intraoperative factors—from the model, as well as the small, single-center sample size. While the current model’s accuracy is insufficient for precise scheduling, it provides a proof-of-concept for ML-based operative time prediction in robotic abdominal wall surgery. Larger, multicenter, prospective studies incorporating surgeon-level and intraoperative variables, as well as comparison with traditional statistical methods, are needed to develop models with sufficient accuracy for clinical implementation. 

## Data Availability

No datasets were generated or analysed during the current study.
